# Troubled Times, Changing Tides: A Seroprevalence Study on Meningococcal Immunity in France Between 2016 and 2024

**DOI:** 10.3390/vaccines13060647

**Published:** 2025-06-16

**Authors:** Samy Taha, Aude Terrade, Oumar Doucoure, Ala-Eddine Deghmane, Muhamed-Kheir Taha

**Affiliations:** Institut Pasteur, Invasive Bacterial Infections, Université Paris Cité, 75015 Paris, France; aude.terrade@pasteur.fr (A.T.); oumar.doucoure@pasteur.fr (O.D.); ala-eddine.deghmane@pasteur.fr (A.-E.D.); mktaha@pasteur.fr (M.-K.T.)

**Keywords:** *Neisseria meningitidis*, invasive meningococcal disease, non-pharmaceutical interventions, seroprevalence, IgG antibodies, serogroups, vaccination

## Abstract

Background/Objectives: In France, non-pharmaceutical interventions (NPIs) implemented to control COVID-19 led to a significant decline in invasive meningococcal disease (IMD) cases. However, a rebound in cases, particularly for serogroups W and Y, was observed after the gradual lifting of NPIs, raising questions about an “immunity gap” due to reduced circulation of the bacteria. During the study period, vaccination against MenC was mandatory from 2018, and vaccination against MenB has been recommended since 2022. Methods: We conducted a retrospective seroepidemiological study using 166 normal sera collected between 2016 and 2024. Anti-*Neisseria meningitidis* IgG levels were quantified by ELISA using purified capsular polysaccharides for serogroups B, C, W, Y, and X. Samples were categorized into three periods: pre-NPIs (n = 72), during NPIs (n = 33), and post-NPIs (n = 61). Statistical comparisons were performed using Kruskal–Wallis tests for non-parametric data. Results: Our results show a significant decline in anti-serogroup B IgG antibody levels after the lifting of NPIs (*p* < 0.0001) in line with reduced circulation. Anti-serogroup C IgG antibody levels increased incrementally (*p* = 0.0003), particularly in those aged 1–4 years, likely reflecting a catch-up in anti-meningococcal C vaccination coverage. Anti-serogroup W IgG antibody levels remained stable, suggesting sustained circulation, but shifted to young children in the post-NPI period, potentially due to a genotypic shift. Anti-serogroup Y IgG antibody levels transiently increased significantly (*p* < 0.0001) during the NPI period but then decreased back after their lifting. Anti-serogroup X IgG antibody levels remained stable, consistent with its low prevalence and the absence of targeted vaccination.

## 1. Introduction

*Neisseria meningitidis* (Nm) is a Gram-negative encapsulated bacterium that is usually carried by approximately 10% of the general population [1], as was the case prior to the COVID-19 pandemic. Invasive isolates of *N. meningitidis* can cross the respiratory epithelial barrier into the bloodstream, resulting in an invasive meningococcal disease (IMD), which is a severe, rapidly evolving systemic disease. The epidemiology of meningococcus is unpredictable. It is influenced by phenotypic factors such as serogroups, environmental factors such as the Harmattan winds in sub-Saharan Africa and the Santa Ana winds in Baja California [2], and genotypic factors due to the wide diversity of circulating isolates of Nm [3]. It may also be linked to viral infections, such as the influenza A virus [4].

In France, since March 2020, the non-pharmaceutical interventions (NPIs) implemented to control the spread of the COVID-19 pandemic (lockdowns, social distancing, and mask wearing) led to a sudden and important reduction in IMD cases across all serogroups and age groups, akin to “pressing a reset button”, mainly involving isolates of hyperinvasive genotypes [3]. The overall carriage rate of Nm also fell during this period [5], as did the compulsory MenC vaccine uptake for the 12-months dose in April 2020 compared to expected levels, in the absence of vaccination recommendations against the other serogroups [6]. These NPIs were gradually lifted from mid-2022. There were two opposing assumptions as to how the epidemiology of IMD would evolve in France. The first was that the reduction in the carriage and circulation of isolates would lead to a sustained reduction in the number of IMDs. Unfortunately, the second hypothesis proved correct, predicting a rapid rebound in the number of cases following the return of circulating isolates in a population rendered naïve by the reduction in both natural and acquired immunity. Since September 2022, there has indeed been a rebound across all serogroups and all age groups, but particularly for serogroups W and Y and among the adolescents and young adults [3]. Corresponding reductions and resurgences of cases occurred in other European countries [7] and also for other respiratory pathogens [8], sometimes after a delay [9].

These post-NPI re-emergences of respiratory pathogen cases were theorized to be due, at least in part, to an “immunity gap”, induced by the lack of immune stimulation following the reduced circulation of these microbial agents and due to reduced vaccine uptake [10]. This notion, also known as an “immune debt”, is controversial and debated [11]. Indeed, to our knowledge, there is only one study providing serological evidence supporting this concept, despite mathematical modelling attempts [12]. This study shows a significant decrease in antibody levels against *Mycoplasma pneumoniae* among children between 2019 and 2023 [13].

The profile of the modification in the number and serogroup distribution of cases of IMD in France, compared with those in other neighboring countries with national vaccination policies [7], led the French National Authority for Health to modify the vaccination calendar. Since 2025, meningococcal B vaccination, which has been recommended for all children under the age of 2 years since 2022, became compulsory and a mandatory vaccination against serogroups A, C, W, and Y replaced the vaccination against serogroup C alone, which has been compulsory since 2018 for all children under the age of 18 months [14].

These evolving vaccination policies, alongside the unprecedented impact of the COVID-19 pandemic and associated NPIs, create a complex landscape for understanding the seroprevalence of *N. meningitidis* in France. This makes a seroepidemiological study an interesting method to better understand the quantitative and qualitative evolution in the number of cases since 2022, in relation to the “immunity gap” phenomenon.

The aim of this study was to evaluate the seroprevalence of anti-*N. meningitidis* IgG in the sera of non-IMD patients for the major serogroups circulating in France (B, C, W, X, and Y), before, during, and since the lifting of the NPIs.

This study focused on those five *N. meningitidis* serogroups due to their distinct epidemiological and immunological profiles in France. Serogroup X, although rare and not targeted by vaccination, continues to cause a few cases of IMD each year, making it a useful reference for low-level immune priming [3]. Conversely, serogroup A has not been reported since the early 2000s and may not be suitable for this study [15]. Serogroup B remains predominant in France, including during the COVID-19 pandemic, and serogroup B capsular antibodies may be used to measure the circulation of serogroup B isolates, as vaccines against this serogroup target subcapsular proteins rather than capsular antigens [3]. Serogroup C, previously the second most frequent, drastically declined after the introduction of mandatory infant vaccinations in 2018 [3]. Serogroups W and Y showed a marked resurgence in 2022 after the NPIs were lifted, despite the absence of national immunization recommendations [3], unlike other European countries that vaccinate adolescents against MenACWY [16,17].

## 2. Materials and Methods

### 2.1. Sample Selection

This study utilized a retrospective design, analyzing serum samples from the National Reference Centre for meningococci and *Haemophilus influenzae* (NRCMHi) collection at Institut Pasteur Paris. A total of 166 sera, corresponding to all available PCR-negative sera for meningococci, which were received between 2016 and 2024 as part of the epidemiological surveillance mission of the NRCMHi, were included. The collection relies on the receipt of samples, and as such, was affected by the COVID-19 pandemic, resulting in a smaller number of samples received during the non-pharmaceutical interventions period. The following data were collected for each serum sample: date of sampling, sex, and age range.

### 2.2. Purification of Meningococcal Capsular Polysaccharides

Capsular polysaccharides from serogroups B, C, W, X, and Y were purified using the same Cetavlon-based method originally described by Nato et al. [18]. All serogroups followed the same protocol: bacterial cultures were grown overnight on Columbia blood agar plates (Merck, Darmstadt, Germany), harvested, and suspended in 0.1 M Tris-HCl buffer (pH 7.4). The cells were disrupted by heating at 56 °C for 30 min to inactivate the autolytic enzymes. After centrifugation, the supernatant was collected and subjected to Cetavlon precipitation by the slow addition of a 1% (*w*/*v*) hexadecyltrimethylammonium bromide solution (Cetavlon), followed by incubation at room temperature for 1 h under gentle agitation to precipitate the capsular polysaccharide. The pellet was collected using centrifugation at 10,000× *g* for 20 min, dissolved in 0.3 M sodium acetate, and then re-precipitated with 3 volumes of cold ethanol. This step was repeated twice to enhance purity. Final purification was achieved by dialysis against distilled water for 48 h with multiple buffer changes, followed by lyophilization. The concentration and purity of the polysaccharides were evaluated using spectrophotometry (A260/A280 ratio) and by performing uronic acid quantification. Only preparations meeting quality standards (A260/A280 < 0.3; low protein/DNA contamination) were used for the ELISA assays.

### 2.3. Quantitation of N. meningitidis Polysaccharide IgG Antibodies (Anti-Nm IgG) Titers Assay

Enzyme-linked immunosorbent assays (ELISAs) was performed on the purified capsular polysaccharides of serogroups B, C, W, Y, and X. ELISA protocols have been described in another study [15]. The results were expressed as optical density values at a wavelength of 450 nm (OD_450_). All samples were above the lower limit of detection value of 0.1. Sera from patients recovering from IMD were used as positive controls and serum from healthy subjects as negative controls. All samples were thereafter normalized using the background value corresponding to the negative controls (i.e., wells of negative sera).

### 2.4. Statistical Analysis

Data were analyzed using GraphPad PRISM 5.0.1 software. The data were grouped into three periods: before NPIs (1 January 2016–12 March 2020; n = 72), during NPIs (13 March 2020–31 August 2022; n = 33), and post NPIs (1 September 2022–30 September 2024; n = 61). As the distribution of the data was not Gaussian for all periods, and to account for the significant variation in the number of samples for each period, Kruskal–Wallis tests for non-parametric data were used to test the differences between groups. Data were also represented by the geometric means with confidence intervals at 95% for each period.

In addition to Kruskal–Wallis tests across time periods, one-way ANOVA was performed by age group for each serogroup using log-transformed OD_450_ values. Bonferroni correction was applied for multiple testing (α = 0.01). Post-hoc differences were assessed using Tukey’s HSD test. Age groups were defined as follows: <1 year; 1–4 years; 5–9 years; 10–14 years; 15–24 years; 25–44 years; 45–64 years; and 65 years and older.

The results of the statistical tests were two-sided, with a *p*-value of <0.05 considered statistically significant.

### 2.5. Ethics Approval

This study was conducted in accordance with the Declaration of Helsinki, and the protocol was approved by the CNIL (Commission Nationale de l’Informatique et des Libertés) N°1475242/2011 on 19 January 2011. The requirement for consent was waived.

### 2.6. Role of Funding Source

This study was funded by the Institut Pasteur. The funder had no role in designing, conducting, analyzing, or writing this study. The study design, data collection, data analysis, data interpretation, and writing of the report were performed by the authors of the study.

## 3. Results

A total number of 166 meningococcus-negative serum samples collected between 2016 and 2024 were analyzed and grouped into three periods. Serogroup-specific anti-*N. meningitidis* IgG levels were measured using an ELISA on purified capsular polysaccharides for serogroups B, C, W, Y, and X.

The age characteristics of the population are presented in Table 1. Sex distribution was nearly equal (84 males [50.6%] and 82 females [49.4%]). The most represented age group was the 1–4 years category (24.1%), followed by those aged <1 year and 45–65 years (16.3% each). Other age groups were distributed as follows: 5–9 years (6.0%), 10–14 years (7.8%), 15–24 years (6.6%), 25–44 years (10.8%), and ≥65 years (12.0%). For each age group, the geometric means (GMs) of the measured OD_450_ values, along with their corresponding 95% confidence intervals, are indicated by serogroup (Table 1). There were no significant differences in the geometric means of the OD_450_ values across the different age groups for any of the serogroups tested.

**Figure 1:**
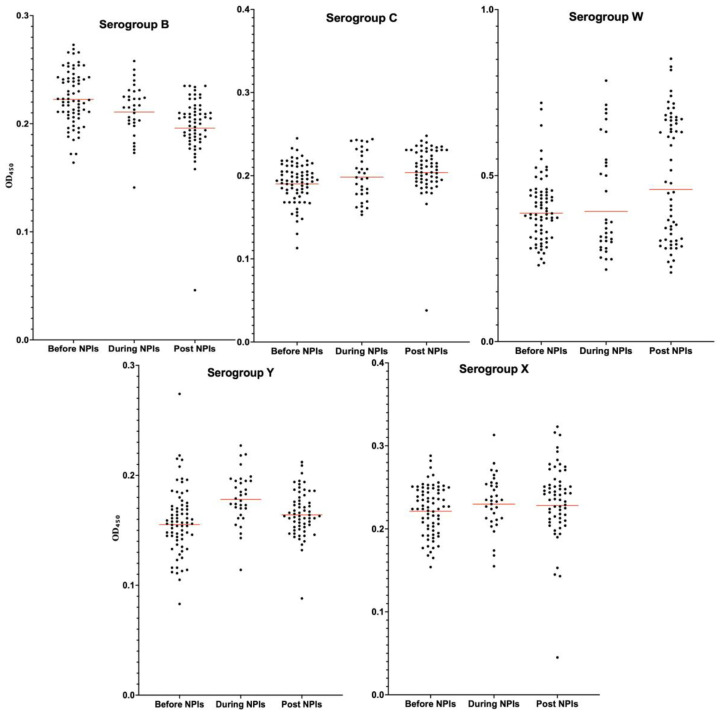
Scatter dot plots of OD_450_ values for five *N. meningitidis* serogroups. The geometric means (GMs) of titer values, with 95% Confidence Intervals, are shown as red lines for each serogroup.

To understand the impact of NPIs and vaccination on population immunity, the results for each serogroup across the defined periods are summarized below and illustrated in Figure 1 with scatter dot plots showing the GM of the measured OD_450_ values, along with their corresponding 95% confidence intervals.

### 3.1. Serogroup X

OD_450_ values for serogroup X were consistently low and stable across all periods. No significant temporal or age-based variation was observed. This result aligns with the epidemiological profile of MenX: rare, low-circulating, and not targeted by vaccination strategies. It also validates our methodological approach, showing that ELISA-measured IgG titers can serve as a proxy for immune priming of the population and MenX can serve as a negative control.

### 3.2. Serogroup C

Although incremental increases in titer values were noted from one period to the next, these changes were not statistically significant except when comparing the pre-NPI and post-NPI periods, where a significant increase was detected (*p* = 0.0003).

Furthermore, post-hoc Tukey analysis revealed significantly higher titers in the 1–4 years group compared to ≥65 years (*p* = 0.0158).

This pattern likely reflects a catch-up in MenC vaccination coverage following its mandatory implementation for toddlers in 2018. Indeed, vaccination coverage for MenC among children aged less than 2 years old in France rose from approximatively 77% in 2019 to almost 90% in 2024 [19].

### 3.3. Serogroup B

A non-significant decline in titer values was observed between the “before NPIs” and “during NPIs” periods (*p* = 0.19). However, titer values further declined significantly in the “Post NPIs” period compared to “during the NPIs” (*p* = 0.04). Overall, the difference between the pre-NPI and post-NPI periods was highly significant (*p* < 0.0001). These findings indicate a persistent reduction in serogroup B seroprevalence in France, despite the lifting of NPIs and changes in vaccination policy in 2022.

### 3.4. Serogroup W

No statistically significant changes in titer values were observed across the three periods. A non-significant upward trend was observed in the post-NPI period relative to the pre-NPI period (*p* = 0.09). This stability suggests the sustained circulation of serogroup W during the study period.

However, the titer population that seemed homogeneous in the before-NPIs period shifted to lower titer values in the during-NPIs period and then again to higher titer values in the post-NPIs period, featuring an hourglass-shaped scatterplot (Figure 1).

We therefore classified the serogroup W titers into two subgroups by comparing them to a threshold represented by the median value (0.65): “high titers” for those above it and “low titers” for those below it. We then compared the distribution by age group for each of these two subgroups using a chi-squared test and found that children aged under 15 were significantly more represented in the “high titers” group. Adults aged over 25 were significantly more represented in the “low titers” group (*p* = 0.002).

**Figure 2:**
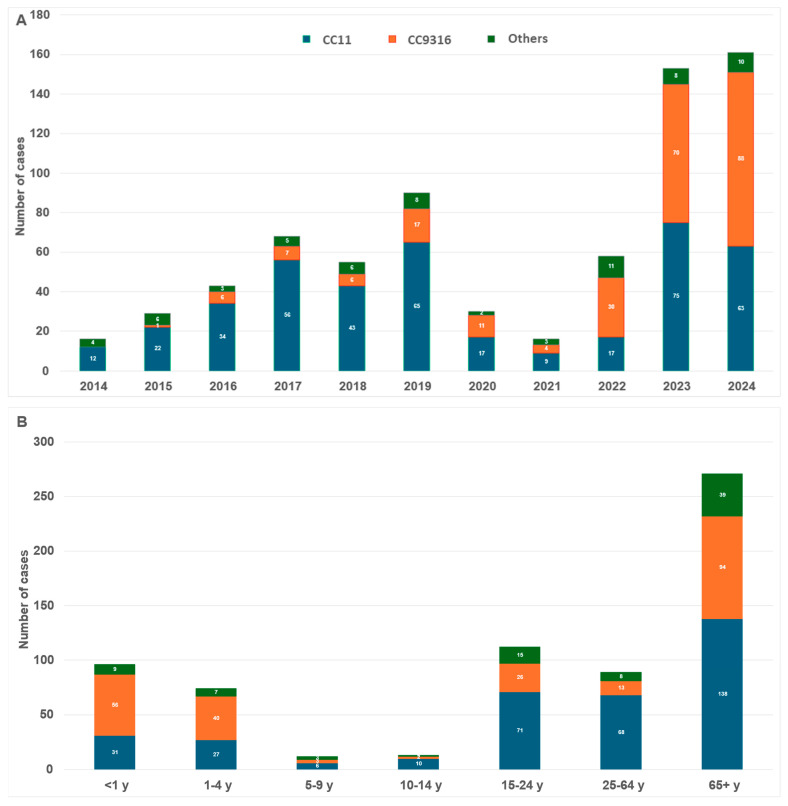
(**A**) Clonal complexes involved in IMDW cases by year, France, 2014–2024. (**B**) Clonal complexes involved in IMDW cases by age group, France, 2014–2024.

This observation could be correlated to the evolutionary dynamics of involved clonal complexes for serogroup W IMD cases, as shown in Figure 2. Indeed, the CC11 hyperinvasive clonal complex experienced a significant decrease in W isolates during the NPI period. Meanwhile W isolates from a newly defined CC9316 clonal complex emerged in 2016 in France and have been responsible for increasing proportions of IMDW cases since. Unlike CC11, it continued to increase in proportion to 30% of IMDW isolates between 2020 and 2022, and rebounded sharply after 2022. As a result, CC9316 was the most involved clonal complex in IMDW in 2022 and 2024 in France, representing 52% and 55% of IMDW isolates for these years, respectively. When stratified by age group, the data shows that CC9316 is highly prominent at both ends of life, as it represented more than half of IMDW cases among children aged less than 5 years old and 35% of IMDW among older adults aged 65 years old and over.

### 3.5. Serogroup Y

A distinct evolution was noted for serogroup Y. During the NPI period, titer values increased significantly (*p* < 0.0001), followed by a significant decrease in the post-NPI period (*p* = 0.02). Although the pre-NPI period exhibited slightly higher titer values compared to the post-NPI period, this difference was not statistically significant (*p* = 0.19). These findings suggest an acceleration of serogroup Y circulation during the pandemic, with a subsequent decline after the NPIs were lifted.

## 4. Discussion

This seroepidemiological study provides insights into the impact of COVID-19-related NPIs on the circulation of different *N. meningitidis* serogroups in France.

For serogroup B, the marked decline in IgG levels from the pre-NPI to the post-NPI period suggests a persistent reduction in the circulation of this serogroup. This observed decline might reflect a decrease in the natural boosting of immunity through asymptomatic carriage during the NPI period when the circulation of serogroup B likely decreased, as suggested by the overall decline in IMD cases. Moreover, the immunogenicity of serogroup B capsular polysaccharides is weak [20], and this reduced exposure could lead to waning IgG levels in individuals. Indeed, age-stratified epidemiological data in France showed that the rebound in the number of cases did not affect all age groups at the same time. It began among the 15–24-year-olds, spared the 65-year-olds, and was over in the first months [3].

The implementation of a vaccination against MenB among children under 2 years of age since 2022, which became mandatory in 2025, aimed to control the rebound of IMD B cases in France, but is not expected to impact the seroprevalence against the MenB capsule as MenB vaccines target subcapsular proteins [21,22,23]. This distinction is crucial, as it means that our ELISA-based measurements of capsule-specific IgG offer a window into natural immune priming independently of recent vaccination.

Our findings align with the commentary of Bloom et al. [24], who emphasize the emergence of an “immunity debt” following the COVID-19 pandemic due to reduced exposure to pathogens, based on the observation of an increase in IMDB cases in the UK and France since restriction measures were eased. The decline in MenB-specific IgG titers observed in our study may reflect this decreased immunological stimulation. Although ELISA titers do not assess protection against disease or carriage, they may reflect exposure to bacterial antigens. Their decline could support the hypothesis that population-level susceptibility may increase in the absence of natural boosting, particularly in the context of waning vaccine-induced immunity or low vaccination coverage in adolescents [25,26,27].

In contrast, serogroup C demonstrated a significant increase in antibody levels when comparing the pre-NPI and post-NPI periods. This increase likely reflects a successful catch-up in vaccination, especially considering that MenC vaccination has been mandatory in France since 2018 [28]. As vaccination coverage improved, the immune response in the population appears to have recovered, despite an overall transient dip during the early pandemic period [6].

The situation with serogroup W is particularly notable. Hadley et al. [29] predicted a long-lasting decline in MenW carriage, driven primarily by pandemic-related reductions in social contacts and by adolescent MenACWY vaccination, which began in 2015. These results contrast with our findings, which show that in France, despite the widespread implementation of NPIs and the absence of routine vaccination recommendations against it until January 2025 (Table 2), IgG levels against serogroup W remained largely unchanged. This difference could suggest that even in the context of reduced bacterial transmission, the existing immune response (either from prior exposure or vaccination) may persist at the population level.

Indeed, the rebound in cases of serogroup W observed in France since the easing of NPIs may reflect a genotypic transition within the historical hyperinvasive clonal complex CC11 for this serogroup, indicating the emergence of a new CC9316 clonal complex (Figure 2)**.** This has also been supported by epidemiological data showing a delayed but significant drop in invasive cases of serogroup W later during the pandemic compared to serogroup B and C [3].

Serogroup Y displayed an unusual pattern. The highly significant surge in IgG levels during the NPI period may reflect an acceleration in the circulation of serogroup Y isolates, a phenomenon that aligns with previous reports of IMD cases—particularly serogroup Y-related respiratory forms—during the first half of 2020 [30]. Indeed, this serogroup is more frequently involved in meningococcal pneumonia [31]. The subsequent decrease in circulation since the end of 2022 suggests a natural restriction of this transient spike for this serogroup. Indeed, serogroup W saw a greater increase in the number of cases in 2023 and 2024, whereas serogroup Y became the second most common cause of IMD cases in France, behind serogroup B, in 2022 [32].

Finally, the stability at low levels observed for IgG against serogroup X over the study period reinforces its characterization as a rare serogroup [33]. Given that it is not targeted by routine vaccination strategies, its consistent seroprevalence likely reflects its low circulation.

The “immunity gap” hypothesis suggests that the reduced exposure to pathogens during NPIs would lead to a decline in population immunity across the whole population, resulting in a subsequent rebound of infections upon the relaxation of restrictions. Although the observed decline in IgG levels for some serogroups during the NPI period might appear consistent with this hypothesis (reflecting reduced natural boosting), the overall picture is more complex. The heterogeneous nature of the seroprevalence trends across the different serogroups underscores that the impact of NPIs and other factors, such as vaccination policies, vaccination coverage, and strain-specific and age-specific characteristics, is not uniform. This complex interplay of factors influencing *N. meningitidis* epidemiology needs to be accounted for when discussing the “immunity debt” concept.

This seroepidemiological study has several limitations. The first is the relatively small sample size of 166 individuals that could lead to a lack of statistical power to detect subtle but important trends. Also, if the samples were selected across different age groups and geographical regions, we did not have access to socioeconomic data of the patients, which could induce bias in the results. Above all, these samples were selected from an active disease surveillance repository and may not be representative of the French population, as shown by the age distribution of our study population (Table 1). Additionally, as this study presents observational data, it cannot definitively establish causal relationships. Finally, this study relies on ELISA to measure IgG antibody levels against *N. meningitidis*. Although ELISA is a widely used and convenient method for quantifying antibodies, its utility as a direct proxy for effective immunity against this pathogen is limited, as it does not directly assess the functional capacity of these antibodies to neutralize or eliminate the bacteria.

## 5. Conclusions

Our findings suggest that NPIs had a significant effect on the seroprevalence of anti-*N. meningitidis* IgG antibodies, with varying trends observed across different serogroups, unlike the uniform decline in immune response theorised by the “immunity debt” concept. These findings support the implementation of mandatory vaccination among children <2 years of age using the 4CMenB vaccine against MenB and the tetravalent ACWY vaccine. Additionally, these vaccines will also be provided to adolescents and young adults [34]. Ultimately, our study highlights the complex interplay between NPIs, vaccination policies, and age and isolate specificities in the circulation of each *N. meningitidis* serogroup, and underscores the need for continued surveillance and for large-scale prospective studies to adapt public health measures to protect against this significant public health threat.

## Figures and Tables

**Table 1:** Characteristics of the study population and geometric mean (GM) of OD_450_ values (with 95% Confidence Intervals) for five *N. meningitidis* serogroups.

Age Group	n (%)	Titer for NmB GM (CI95%)	Titer for NmC GM (CI95%)	Titer for NmW GM (CI95%)	Titer for NmY GM (CI95%)	Titer for NmX GM (CI95%)
<1 year	27 (16.3)	0.2043	0.2067	0.6394	0.1681	0.2363
		[0.1952; 0.2137]	[0.1964; 0.2174]	[0.6165; 0.6632]	[0.1606; 0.176]	[0.2208; 0.2529]
1–4 years	40 (24.1)	0.2149	0.2087	0.6425	0.1625	0.2386
		[0.2086; 0.2214]	[0.2011; 0.2166]	[0.6126; 0.6739]	[0.1523; 0.1733]	[0.23; 0.2476]
5–9 years	10 (6.0)	0.2134	0.1948	0.6575	0.17	0.2226
		[0.193; 0.2359]	[0.1787; 0.2124]	[0.6223; 0.6946]	[0.1599; 0.1808]	[0.1988; 0.2494]
10–14 years	13 (7.8)	0.2197	0.1993	0.6861	0.1685	0.2351
		[0.2049; 0.2356]	[0.185; 0.2148]	[0.6358; 0.7405]	[0.149; 0.1904]	[0.2156; 0.2563]
15–24 years	11 (6.6)	0.2042	0.1899	0.6294	0.1638	0.2213
		[0.1873; 0.2226]	[0.1752; 0.2059]	[0.5912; 0.67]	[0.1516; 0.1769]	[0.2003; 0.2445]
25–44 years	18 (10.8)	0.1937	0.179	0.616	0.1267	0.195
		[0.1606; 0.2337]	[0.1466; 0.2187]	[0.5712; 0.6643]	[0.08167; 0.1967]	[0.16; 0.2376]
45–65 years	27 (16.3)	0.2183	0.1948	0.6514	0.1606	0.2227
		[0.2075; 0.2296]	[0.1856; 0.2044]	[0.6278; 0.6759]	[0.1497; 0.1723]	[0.2084; 0.238]
≥65 years	20 (12.0)	0.2085	0.1827	0.6141	0.1615	0.217
		[0.1961; 0.2216]	[0.1698; 0.1966]	[0.5642; 0.6685]	[0.1502; 0.1736]	[0.204; 0.2308]

**Table 2:** Vaccination programs among infants aged <2 years of age during each of the study periods.

Vaccination	Pre-NPIs	NPIs	Post-NPIs	Since January 2025
NmC	Mandatory since 2018	Mandatory	Mandatory	No
NmACWY	No	No	No	Mandatory *
NmB	No	No	Recommended since 2022	Mandatory *

* Vaccination against MenB and ACWY was also recommended for adolescents and young adults. NPIs: non-pharmaceutical interventions.

## Data Availability

The original contributions presented in this study are included in the article. Additional data presented in this study are available on request from the corresponding author due to privacy restrictions.
